# The application of tDCS in psychiatric disorders: a brain imaging view

**DOI:** 10.3402/snp.v6.29588

**Published:** 2016-03-17

**Authors:** Chris Baeken, Jerome Brunelin, Romain Duprat, Marie-Anne Vanderhasselt

**Affiliations:** 1 Department of Psychiatry and Medical Psychology, Ghent University Hospital, Ghent University, Ghent, Belgium; 2 Department of Psychiatry University Hospital (UZBrussel), Brussels, Belgium; 3 Ghent Experimental Psychiatry (GHEP) Lab, Ghent, Belgium; 4 INSERM U1028, CNRS UMR5292, PSYR2 Team, Lyon Neuroscience Research Center, Centre Hospitalier Le Vinatier, Université Claude Bernard Lyon 1, Villeurbanne, France; 5 CIRRIS-Centre Interdisciplinaire de Recherche en Réadaptation et en Intégration Sociale, Université Laval, Québec, Canada; 6 Faculty of Medicine and Pharmacy, Free University Brussels, Brussels, Belgium

**Keywords:** brain imaging, neuromodulation, neurostimulation, psychiatric disorders, tDCS

## Abstract

**Background:**

Transcranial direct current stimulation (tDCS) is a non-invasive, non-convulsive technique for modulating brain function. In contrast to other non-invasive brain stimulation techniques, where costs, clinical applicability, and availability limit their large-scale use in clinical practices, the low-cost, portable, and easy-to-use tDCS devices may overcome these restrictions.

**Objective:**

Despite numerous clinical applications in large numbers of patients suffering from psychiatric disorders, it is not quite clear how tDCS influences the mentally affected human brain. In order to decipher potential neural mechanisms of action of tDCS in patients with psychiatric conditions, we focused on the combination of tDCS with neuroimaging techniques.

**Design:**

We propose a contemporary overview on the currently available neurophysiological and neuroimaging data where tDCS has been used as a research or treatment tool in patients with psychiatric disorders.

**Results:**

Over a reasonably short period of time, tDCS has been broadly used as a research tool to examine neuronal processes in the healthy brain. tDCS has also commonly been applied as a treatment application in a variety of mental disorders, with to date no straightforward clinical outcome and not always accompanied by brain imaging techniques.

**Conclusion:**

tDCS, as do other neuromodulation devices, clearly affects the underlying neuronal processes. However, research on these mechanisms in psychiatric patients is rather limited. A better comprehension of how tDCS modulates brain function will help us to define optimal parameters of stimulation in each indication and may result in the detection of biomarkers in favor of clinical response.

Transcranial direct current stimulation (tDCS) is a recently reintroduced, non-invasive, superficial, and non-convulsive technique that can modulate brain function. It usually involves the application of a weak current (0.5–2 mA) between an anode and a cathode, which are placed on specific chosen locations over the human scalp (Fig. 1). Most tDCS studies use saline-soaked sponges or conductive gel electrodes for stimulation (usually between 25 and 35 cm^2^), resulting in current densities at the scalp surface of up to ~0.08 mA/cm^2^ (Johnson et al., 2013). The cortical excitability is polarity dependent: increases in neuronal excitability occur under the anode (by slightly depolarizing the membranes), and a decrease is observed under the cathode (hyperpolarizing neurons’ membranes) (Nitsche, Liebetanz, et al., 2003). Besides size, polarity, and position of the electrodes, the applied current intensity, density, duration of stimulation, and the properties of the tissue in the stimulated areas may influence the neurobiological effects of tDCS (Medeiros et al., 2012). Even though increasing the current density and duration of stimulation can lead to more significant and longer-lasting effects on cortical activity, computational modeling has shown that it is important to maintain relatively weak currents in order to retain the subthreshold effects of tDCS on cortical excitability and to avoid safety concerns with higher levels of electricity (e.g. Parazzini, Fiocchi, Rossi, Paglialonga, & Ravazzani, 2011).

**Fig. 1:**
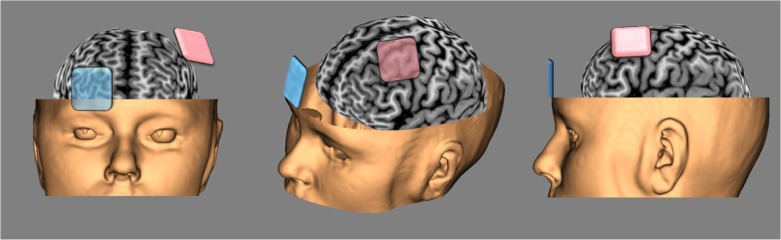
Typical tDCS montage for cognitive/emotional research, that is, for the treatment of Major Depressive Disorder (anode in pink over the left DLPFC, cathode in blue over the right orbitofrontal cortex).

Computational studies modeling the electric field distribution induced by tDCS (for a review see Miranda, Mekonnen, Salvador, & Ruffini, 2013) are important to understand how circuitries are being rearranged and functioning is adapted (i.e. neuroplastic changes). Besides verifying the broad effect induced by typical stimulation electrodes, modeling studies observed that in a usual montage (two electrodes on the skull), the maximum of the electric field is not necessarily directly underneath the anode and cathode but also lies in the cortical and subcortical areas between the two electrodes (Salvador, Mekonnen, Ruffini, & Miranda, 2010). See also Fig. 1.

Compared to other non-invasive brain stimulation techniques (e.g. electroconvulsive therapy – ECT, repetitive transcranial magnetic stimulation – rTMS), where costs, clinical applicability, and availability limit the large-scale use in clinical practices, the low-cost, portable and easy-to-use tDCS devices may overcome these restrictions. In addition, a tremendous number of studies have shown that anodal tDCS, particularly applied over the dorsolateral prefrontal cortex (DLPFC), can improve cognitive functions and emotional processes (e.g. Fregni et al., 2006). The enhancement of cognitive functioning is usually based on a single session stimulation protocol, and these effects are transient. Multiple sessions on the contrary seem to produce longer-lasting effects. Non-invasive brain stimulation techniques such as tDCS are increasingly studied as potential adjunct therapies for a wide range of psychiatric disorders (e.g. Mondino et al., 2014; Tortella et al., 2015). However, before common use at home can be expected (as it is easy in its application), its neurophysiological effects on the human brain remain to be determined. Not only in terms of safety and ethical issues, or concerning the neural correlates effects of neuromodulation, but also regarding the exact working mechanisms. Furthermore, individual differences in the neuroanatomy of the brain and the genetic polymorphism (e.g. brain derived neurotrophic factor, BDNF) are important to consider when looking at the neuronal and clinical effects of tDCS (Chew, Ho, & Loo, 2015). On the contrary, because abnormalities in brain activity, plasticity, and functional connectivity have been identified as potential underlying causes in many psychiatric diseases (Kuo, Paulus, & Nitsche, 2014), tDCS might have an impact on functional cortico-subcortical networks involved in the respective mental illnesses (Polania, Paulus, & Nitsche, 2012).

All in all, the side effects of the tDCS technique are reported to be limited in experimental as well as in clinical trials (Nitsche, Liebetanz, et al., 2003), and in a variety of neurological and psychiatric disorders the therapeutic efficacy seems promising (Brunoni et al., 2012; Mondino et al., 2014). Given that tDCS is a relatively new therapeutic tool, its potential clinical benefits are limited by the low number of studies. Even though a better understanding of its neurophysiological working mechanisms may be necessary to guide and improve future tDCS treatment protocols, so far, brain imaging studies in psychiatrics are rather scarce. Therefore, in order to present potential directions for future research, we aim at providing a contemporary overview of the use of tDCS in combination with brain imaging techniques in the healthy as well as in the ‘mentally’ affected human brain. We will start with a general overview of tDCS research using brain imaging, to further illuminate research in mental disorders.

## tDCS and brain imaging

To investigate the electrical field modulation *in vivo*, without invasive neurosurgery, brain imaging techniques allow studying neural changes in the brain. Electrophysiological (such as electroencephalography: EEG with specific temporal resolution) and neuroimaging (such as functional magnetic resonance imaging: fMRI, with specific spatial resolution) methods provide crucial information regarding the neural activity of specific brain areas and/or circuitries targeted by the neuromodulation in the living humans. Brain imaging studies have also assessed the effects of tDCS in animal models (e.g. Joy, Lebedev, & Gati, 1999). These biological techniques have the advantage of measuring correlates of neuronal activity not only under the close proximity of the external applied electrodes but also in more remote cortico-subcortical brain neurocircuits. The trans-synaptic spreading will depend on the strength and level of activity of brain networks. Besides the effects of the electrical tDCS fields on brain networks, at the cellular level, the applied external electric field is able to modify transmembrane potential differences by forcing displacements of intracellular ions modifying spike firing probability measured with intracellular and voltage-sensitive dye recordings (Bikson et al., 2004). Related to stimulation duration, synaptic driven after-effects are induced, depending on the individual neural morphology, the connected pathways and the orientation of the somatodendritic axis (Krause, Márquez-Ruiz, & Cohen Kadosh, 2013). In addition, motor evoked potential (MEP) studies showed that anodal and cathodal tDCS after-effects are influenced by glutamatergic and GABAergic neurotransmissions (Nitsche, Fricke, et al., 2003, 2004). Importantly for its application in psychiatric disorders, the initial effects of tDCS to induce neuronal depolarization or hyperpolarization may result in lasting effects characterized by long-term potentiation (LTP)- and long-term depression (LTD)-like effects (Paulus, 2004). Indeed, Paulus (2004) reports that tDCS induces a permanent change in the excitability of nerve cells characterized by mechanisms similar to LTP and LTD, which are the manifestation of a change in N-Methyl-D-aspartate (NMDA) receptor activity. This results changes in neuroplasticity which depend on the stimulus duration and intensity. Liebetanz, Nitsche, Tergau, and Paulus (2002) stated in their MEP study that the induction of the tDCS-induced after-effects requires a combination of glutamatergic and membrane mechanisms, similar to the induction of established types of short or long-term neuroplasticity.

There have only been few attempts to simultaneously record EEG during tDCS stimulation (as an example, we refer to Faria, Fregni, Sebastiao, Dias, and Leal (2012) studying epileptic patients). Because EEG has high temporal resolution, the co-registration will provide information regarding the temporal effects of neuromodulation on electrical field distributions. A recent study applying online EEG observed that the neural changes are rapid and persist a couple of minutes after the tDCS has ended (Accornero et al., 2014), but reports in psychiatric patients are non-existent so far. A recent study combining simultaneously tDCS with magnetoencephalography (MEG) reported that tDCS modulated slow cortical magnetic field in brain regions that precisely matched prior metabolic neuroimaging studies (Garcia-Cossio et al., 2015).

To conclude, only 2 decades ago, the simultaneous application of tDCS and neuroimaging methods were considered unfeasible (e.g. heating under the electrodes, quality of the acquired data). In the last couple of years, it became technically feasible and safe to simultaneously stimulate the brain and measure the blood flow in an MRI environment. Although research on the co-registration of fMRI and tDCS is currently flourishing, and will be very important to illuminate the spatial connectivity patterns following tDCS, clear results are still scarce. For instance, confirming neurophysiological studies, while undergoing Arterial Spin Labeling (ASL) in the MRI scan, it has been reported that that anodal tDCS induced an increase in regional cerebral blood flow (rCBF) during and after the stimulation period, whereas cathodal tDCS resulted in a decreased rCBF after stimulation but an increased rCBF during the stimulation period (Zheng, Alsop, & Schlaug, 2011). These effects were not only observed in the brain regions under the electrodes but also in other brain areas along a network of brain regions that are functionally related to the stimulated area (Zheng et al., 2011).

Finally, it is important to note that the tDCS application not only modulates synaptic connectivity but it also induces neuroplastic changes regulated by several neurotransmitters systems including dopamine, acetylcholine, and serotonin, BDNF. The tDCS application also affects neuronal membrane channels, such as sodium and calcium pumps. Because this is beyond the scope of this review, we refer the interested reader to the correct literature (Fritsch et al., 2010; Medeiros et al., 2012; Ruffini et al., 2013).

## tDCS in psychiatric disorders

Given that psychiatric disorders demonstrate (as compared to controls) disrupted functional and structural neural networks, from a clinical perspective, the tDCS application may modulate functional connectivity and induce synchronization changes. So far, however, neuroimaging evidence of the distributed network modulatory effects of tDCS is largely limited to the motor system. In the following paragraphs, we will focus on research conducted in different psychiatric disorders, specifically focusing on recent trends and new directions in the field.

## Major depressive disorder (MDD)

Major depressive disorders (MDD) are highly prevalent and are associated with serious personal suffering and societal costs (Kessler et al., 2010). MDD is primarily characterized by persistent low mood, recurrent negative thoughts and anhedonia. Abnormalities in cortico-subcortical circuits are found to be a vulnerability factor for relapse in MDD (De Raedt & Koster, 2010). Abnormal functioning of such neurocircuits may lead to refractory or treatment-resistant conditions (De Raedt, Vanderhasselt, & Baeken, 2015). In psychiatry research, most clinical tDCS studies conducted so far are dedicated to the treatment of major depression, mostly the treatment of unipolar MDD. Few studies examined the possible role of tDCS in bipolar depression and mania, with some indication of its use, but studies were on small samples and usually not accompanied by brain imaging measurements (Brunoni et al., 2011; Schestatsky et al., 2013).

One reason for this extended research in MDD might be that reduced activity in the DLPFC, which is located at the convexity of the brain, provides optimal prerequisites for successful stimulation interventions. Indeed, similar to rTMS, in MDD the anodal tDCS electrode targets the DLPFC. This neocortical area is implicated in regulating affective states, providing cognitive control over stress and emotion responsiveness and is thought to be hypoactive during depressive episodes (Davidson et al., 2002). Decreased neuronal activities in the (dorsolateral) prefrontal regions, as well as in the rostral anterior cingulate cortex (ACC) areas, closely connected to the DLPFC, are often reported (Mayberg, 2003). These frontal hypoactivities result in apathy, psychomotor slowness, and impaired executive functioning. Besides dysfunctional ‘fronto-cingulate networks’, other neuronal pathways between the orbital and medial prefrontal cortex, the subgenual ACC, the amygdala, and hippocampus are implicated as well in the pathophysiology of mood disorders (Baeken & De Raedt, 2011).

To modulate these neural circuits implicated in MDD, therapeutic strategies of non-invasive brain stimulation techniques have mostly focused on enhancing left DLPFC activity and LTP-like plasticity, and/or decreasing right DLPFC activity (Baeken & De Raedt, 2011). Most of the current tDCS treatment protocols in MDD patients place the excitability-enhancing anodal tDCS over the left DLPFC, with the cathodal electrode positioned over the contralateral supraorbital region (See Fig. 1). Using these electrodes position, most double-blinded, sham-controlled studies successfully apply anodal tDCS over the left DLPFC for around 20 min for 5–15 consecutive days with a stimulation intensity at 2 mA. Weaker and less frequent stimulation – in more severe MDD patients – seem to have inferior outcomes in reducing clinical symptoms (See Kuo et al., 2014; Mondino et al., 2014).

Based on brain models where not only a left hemispheric hypoactivity but also a right hemispheric hyperactivation is observed during depressive episodes, some researchers applied bifrontal tDCS with the anode over the left and the cathode on the right DLPFC in order to re-establish the balance between both hemispheres. Although some open label studies applying bifrontal tDCS were found to be clinically effective (Brunoni et al., 2011; Dell'Osso et al., 2012), one double-blinded sham-controlled study was not (Blumberger, Tran, Fitzgerald, Hoy, & Daskalakis, 2012). Nevertheless, an important large double-blinded sham-controlled bifrontal tDCS study (*n*=120), with or without combination with sertraline, demonstrated that not only tDCS alone improved depression ratings to a similar extent as antidepressant medication, but the combination of tDCS and sertraline obtained superior effects on depressive symptoms (Brunoni et al., 2013). This is interesting as it underscores the importance of tDCS as an add-on treatment to existing pharmacological protocols.

No studies so far have investigated neural changes in fronto-cingulate-limbic neural functioning following tDCS treatment in psychiatric patients. The effects of tDCS have been investigated on resting state brain functioning, overall in healthy volunteers. Resting-state functional MRI has been widely used in depression research. For example, Anand, Li, Wang, Lowe, and Dzemidzic (2009) found that during rest fMRI, functional connectivity between the ACC, limbic system, and the thalamic area was significantly reduced in patients with depression, suggesting abnormalities in resting state cortico-limbic connectivity. Another resting-state fMRI imaging study of Keeser et al. (2011) investigated whether frontal tDCS could increase functional connectivity in networks that include the DLPFC. These researchers performed a placebo-controlled double blind repeated measures study in which a group of 13 healthy participants received both 20 min of real (i.e. 2 mA with the anode over the left DLPFC and the cathode over the right supraorbital region) and sham stimulation. Before and after the stimulation (real or sham) participants underwent a resting state scanning for about 5 min. Active tDCS enhanced functional connectivity in the frontal and fronto-parietal regions (Keeser et al., 2011), regions that are known to play an important underlying role in depression (Price & Drevets, 2012). Peña-Gómez et al. (2012) demonstrated that anodal tDCS over the left and right DLPFC (reference over the contralateral supraorbital site) resulted in an altered temporal functional connectivity between prefrontal and parietal neural networks. More specifically, anodal tDCS induced increased synchrony within the anti-correlated network (AN, strong negative activity correlation with the Default Mode Network (DMN)), whereas neuromodulation reduced these temporal neural correlations in components of the DMN. In other words, these data demonstrate that above and beyond the influence of tDCS on cortical excitability, tDCS provokes widespread alterations in neural synchronization in neural regions implied in the DMN. Moreover, using whole-brain ASL Stagg et al. (2013) demonstrated that anodal tDCS of the left DLPFC resulted in decreases in widespread cortical perfusion (after as compared to during the stimulation) similar to that of the DMN. Finally, an EEG study of Miller, Berger, and Sauseng (2015) demonstrated that tDCS (anode on the AFz, cathode placed underneath the chin) enhanced fronto-theta midline amplitude in a resting EEG condition, which was then associated to neural activity in the frontal and left medial prefrontal brain areas. Frontal–midline theta rhythm (4–8 Hz) has been associated with working memory and allocation of sustained attentional resources. Interestingly, frontal theta EEG activity correlates negatively with the DMN in resting state (Scheeringa et al., 2008).

The above-mentioned tDCS results suggest that an increase in cortical excitability within the DLPFC induced by anodal tDCS leads to a subsequent disturbance of the integrity of the DMN. Possibly, tDCS-induced deactivations of the DMN may prompt or facilitate reallocation of cerebral resources to support task performance, and thereby beneficially influence the regulation of cortico-subcortical network activity. This influence on activity in the DMN should be investigated further in order to understand the effects of anodal and cathodal tDCS on neural circuitries that are importantly implicated in mood disorders. Therefore, further research should investigate the functionality and connectivity of the anterior neural circuitries (DLPFC – ACC and DLPFC – orbitofrontal cortex (OFC)) underlying the effects of tDCS (Weber, Messing, Rao, Detre, & Thompson-Schill, 2014).

## Schizophrenia

Schizophrenia is a chronic psychotic disorder characterized by dysfunctions of perception of reality, emotion, and cognition. Patients may experience positive symptoms, such as hallucinations, delusions, and manifest odd behaviors. Furthermore, negative symptoms may be present in any stage of the disease (i.e. affective flattening, depression-like symptoms, alogia, attentional and motor impairments).

**Fig. 2:**
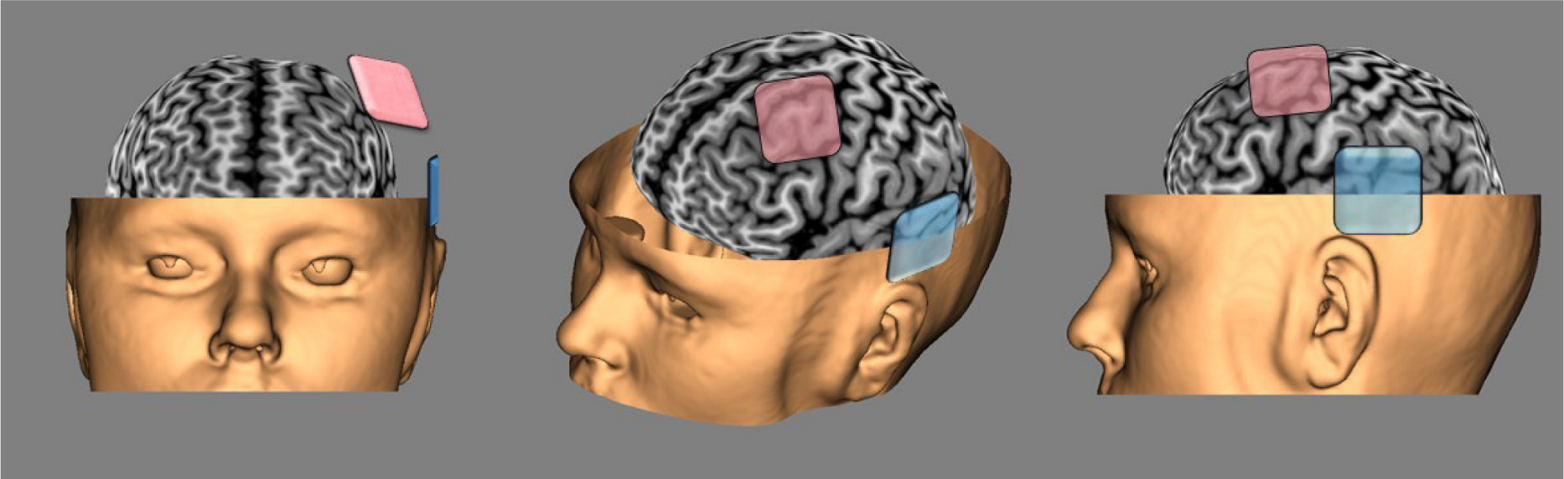
Typical tDCS montage for research and treatment of Schizophrenia (anode in pink over the left DLPFC, cathode in blue over the left temporo-parietal junction).

The primary indication for tDCS in these kinds of patients is to reduce auditory verbal hallucinations. Even when patients are stabilized by antipsychotic medication, this is a frequently observed and persistent symptom in schizophrenia. To eliminate or to reduce these debilitating residual symptoms, the inhibition of neuronal activity related to this hearing of voices may be the inhibition of the left temporo-parietal junction (TPJ) (Brunelin et al., 2012). See Fig. 2.

Low-frequency rTMS applied over the left hyperactive TPJ has also been successfully used in this indication (Lefaucheur et al., 2014). Homan et al. (2011) observed with ASL that after 10 consecutive daily sessions with 1 mA intensity for 15 min cathodal tDCS over the left TPC coupled with anodal tDCS over the right supraorbital region reduced auditory hallucinations by 60% in a psychotic patient. Importantly, this reduction was accompanied by an attenuation of the regional cerebral blood flow measured by ASL under the cathode after each tDCS session. By using a bipolar stimulation approach in a sham-controlled, double-blinded design, Brunelin et al. (2012) investigated in a well-defined sample of schizophrenic patients the efficacy of tDCS on auditory verbal hallucinations and concomitantly on negative symptoms. The authors observed beneficial acute effects on negative symptoms together with a significant acute and prolonged (up to 3 months) reduction of auditory verbal hallucinations. In a sample of patients with schizophrenia partially overlapping the first study, the same group of researchers reported that the reduction of hallucinations severity following active tDCS correlated with a decrease of the functional connectivity fMRI between the left TPJ and the left anterior insula (Mondino, Jardri, et al., 2015). They also reported that compared to sham, active tDCS reduced resting state functional connectivity of the left TPJ with the right inferior frontal gyrus and increased resting state functional connectivity of the left TPJ with the left angular gyrus, the left DLPFC and the precuneus, regions that have been involved in language-related and self-other recognition networks.

In their case report using bifrontal tDCS over the both DLPFCs in a schizophrenic patient, Palm et al. (2013) found that reduction of depressive, positive, and negative symptoms resulted in reduced functional connectivity in the anterior part of the DMN after treatment.

In a series of MEP studies conducted by the group of Hasan and colleagues in patients with schizophrenia (Hasan et al., 2011; Hasan, Aborowa, et al., 2012; Hasan, Nitsche, et al., 2012), anodal and cathodal tDCS neurophysiological effects have been observed, respectively, diminishing excitability enhancement and neuroplasticity. It has been suggested that since tDCS-induced cortical plasticity is dependent on NMDA receptors and is modulated by dopaminergic transmission; this observation can be explained by an imbalance of glutamatergic and dopaminergic systems present in schizophrenia (Javitt, 2010). Indeed, on the neurobiological level, schizophrenia has been associated with dysregulation of several neuromodulatory neurotransmitter systems, such as dopamine, consequently leading to pathological alterations of cortical activity and plasticity (Kuo et al., 2014). In a recent review, Tortella et al. (2015) suggested that cathodal tDCS applied to the TPC may induce LTD-like phenomena, given the decrease in auditory hallucinations, whereas anodal tDCS over the left DLPFC may induce LTP-like phenomena, supported by improvements in negative symptoms. However, the direction of induced changes in neuroplasticity in patients with schizophrenia may also be influenced by external and internal factors such as nicotine smoking (Brunelin et al., 2015) and catechol-O-methyltransferase COMT val158 Met polymorphism (Shivakumar et al., 2015).

One of the few attempts to investigate tDCS induced neural changes in schizophrenic patients, Hoy, Bailey, Arnold, and Fitzgerald (2015) measured EEG oscillations following different tDCS protocols. They reported a significant increase in gamma synchronization (which is proposed to be associated with GABA impairments) in the DLPFC following active tDCS. This was specifically for the 2mA tDCS over the left DLPFC protocol (as compared to the 1 mA and sham protocols), and in the context of improved working memory performance. *This latter study presents data showing tDCS to be able to modulate neural synchrony and thereby restore neural functioning and behavior in schizophrenia*.

## Anxiety disorders and obsessive compulsive disorder (OCD)

Although anxiety disorders comprise a large part of psychiatric conditions, non-invasive brain stimulation techniques have not been able to yield significant beneficial clinical outcomes (Lefaucheur et al., 2011). Further, with the exception of obsessive-compulsive disorder (OCD), no randomized sham-controlled studies yet examined the effect of tDCS treatment in such patients (Bation, Poulet, Haesebaert, Saoud, & Brunelin, 2015; Mondino, Haesebaert, et al., 2015; Narayanaswamy et al., 2014; Volpato et al., 2013). The effects of tDCS in a treatment-resistant case of OCD have been examined on affective symptoms and resting state fMRI activity (Volpato et al., 2013). Even though tDCS did not influence obsessive-compulsive symptoms, it beneficially influenced accompanying depression and anxiety symptoms. This change in symptomatology was associated with an adjustment of the inter-hemispheric imbalance which was observed at baseline (hyperactivation of the left and hypoactivation of the right anterior neural circuits).

## Substance-related and addictive disorders

Substance use disorder is a chronic relapsing disorder and even when patients are effectively detoxified, they often relapse. An important factor contributing to relapse is substance craving (Heinz et al., 2005). Alcohol and drugs of abuse mediate their rewarding effects through the mesocorticolimbic system that consists of the ventral tegmental area (VTA), nucleus accumbens (NAcc), amygdala and prefrontal cortex (Bauer et al., 2013). It is related to abnormal reinforcement of the brain reward circuitry, and prefrontal cortical networks, including the DLPFC, exert a crucial role in inhibitory control mechanisms involved in substance use disorder (Bechara, 2005). Applying right-anodal coupled with left-cathodal tDCS over the bilateral DLPFC have been shown to reduce a variety of substance cravings in patients with substance use disorders (Boggio et al., 2008, 2009, 2010; Fecteau et al., 2014; Fregni, 2008; Klauss et al., 2014). Further it has been suggested that bilateral stimulation with both polarities may be equally effective (Kuo et al., 2014), but much more clinical works are needed to substantiate not only the optimal parameters for tDCS but larger follow-up studies are needed given the high relapse rates among these kinds of patients. The use of event related potentials (ERP) may provide a fast indication whether the neuronal network of interest is targeted. For example, recently detoxified alcoholic patients employ more neural resources than controls, as shown by a specific ERP (e.g. increased P3), when correctly inhibiting a response, and this increase in neural effort predicts relapse 3 months later (Petit et al., 2014). The effects of anodal tDCS of the frontal cortex on P300 have been demonstrated in prior research in alcoholic patients (Nakamura-Palacios et al., 2012). It was recently shown that tDCS of the right inferior frontal cortex specifically decreases the amplitude of the P300 amplitude associated with correctly inhibited responses (Campanella et al., Submitted for publication). These results suggest that tDCS enhances the ability to successfully inhibit a potent response. This is of clinical importance as impaired inhibitory control seems to play a key role in triggering relapse in some pathological states such as addictions. Finally, it has been shown, albeit only in healthy subjects, that prefrontal tDCS may alter activation and connectivity in cortico-subcortical regions. This activation was related to the reward system, including the DLPFC, ACC and OFC (Weber et al., 2014), which plays an important role in vulnerability to substance use disorders.

In eating disorders, food craving being a core symptom originating from a malfunctioning of the lateral prefrontal circuit, studies have shown that tDCS over the DLPFC (anode over the right prefrontal cortex and cathode over the left prefrontal cortex) was effective to reduce food craving (Fregni et al., 2008; Val-Laillet et al., 2015). With the same electrode montage, it has been reported that tDCS over the DLPFC reducing food craving modulated ERP components associated with inhibitory control (N2 and P3a; Lapenta, Sierve, Coutinho de Macedo, Fregni, & Boggio, 2014). As these latter authors did not find any effect on motivational components, this suggests that the reduction in food intake is primarily related to an increased inhibitory control that results from active neuromodulation.

*All in all, brain imaging data could be used to predict tDCS treatment outcome and/or to decide for treatment adjustment in addictive disorders*.

## Other psychiatric disorders

As anodal tDCS is often performed over the left DLPFC, the expected increases in neuronal activity may also influence impaired cognitive functions in a positive way, in particular in patients suffering from Attention-deficit/hyperactivity disorder (ADHD) and autism spectrum disorder (Demirtas-Tatlidede, Vahabzadeh-Hagh, & Pascual-Leone, 2013). Indeed in autistic children, a single session of anodal tDCS over the DLPFC was sufficient to increase peak alpha frequency which was significantly associated with symptom improvement (Amatachaya et al., 2015).

Given its potential role in cognitive enhancement, it is not surprising that clinical studies support the use of tDCS for dementia disorders (Elder et al., 2015; Hansen, 2012). As Alzheimer disease (AD) is associated with an altered temporal correlation in parietal and prefrontal EEG oscillations (Montez et al., 2009), tDCS could be used for therapy as it seems to reconfigure cerebral networks and change functional brain synchronization (e.g. Keeser et al., 2011; Peña-Gómez et al., 2012; Polanía, Nitsche, & Paulus, 2011).

Because tDCS shows efficacy in modulating various cognitive functions, the list of possible clinical application keeps getting longer: dyslexia, Tourette syndrome and PTSD among others could benefit from the development of tDCS as a therapeutic tool. Nevertheless, still relatively few (brain imaging) studies have examined the effects of tDCS in mental disorders, and its efficacy has not yet been proven so far.

## Conclusions

Although the tDCS application has to be considered still in its growing stages, current findings already suggest clinical windows for treatments in a variety of psychiatric conditions. The fact that tDCS can influence certain neural circuits is of crucial clinical importance as abnormal brain activity, plasticity and functional connectivity have been identified as probable underlying causes in many psychiatric diseases (e.g. Uhlhaas & Singer, 2010). *These neurobiological mechanisms also have the potential for extending our understanding of the effects of tDCS regarding the complex relationship between behavioral data and brain topography (e.g. spatial properties of neuroplastic alterations) beyond the stimulated area*. Brain imaging studies point indeed to affecting specific implicated neural circuits connected with the areas where the electrodes are located. *Therefore, combined tDCS research with neuroimaging may not only be able to elucidate the underlying pathophysiology of mental disorders, but it may also be used as guidance to improve tDCS treatment protocols*. Moreover, neuroimaging will also become a non-invasive tool to track for functional recovery and to correlate these changes with behavioral improvements that can predict this recovery. Therefore, tDCS research will undoubtedly play a crucial role in this evolution in the domain of clinical affective neuroscience, albeit a lot of experimental and clinical research needs to be done.
